# Energy-Based Unified Models for Predicting the Fatigue Life Behaviors of Austenitic Steels and Welded Joints in Ultra-Supercritical Power Plants

**DOI:** 10.3390/ma17102186

**Published:** 2024-05-07

**Authors:** Jeong Ho Hwang, Dae-Woong Kim, Jae-Yong Lim, Seong-Gu Hong

**Affiliations:** 1 Institute of Future Energy Technology, FNC Technology Co., Ltd., Yongin 17084, Republic of Korea; jhhwang@fnctech.com; 2 Convergence Research Center for Meta-Touch, Korea Research Institute of Standards and Science, Daejeon 34113, Republic of Korea; kimdw@kriss.re.kr; 3 Division of Chemical and Material Metrology, Korea Research Institute of Standards and Science, Daejeon 34113, Republic of Korea; 4 Department of Safety Engineering, Seoul National University of Science and Technology, Seoul 01811, Republic of Korea; 5 Department of Nano Science, University of Science and Technology, Daejeon 34113, Republic of Korea

**Keywords:** ultra-supercritical power plant, boiler tube material, austenitic steel, low-cycle fatigue, fatigue life prediction model, plastic strain energy density, elevated temperature

## Abstract

The development of a cost-effective and accurate model for predicting the fatigue life of materials is essential for designing thermal power plants and assessing their structural reliability under operational conditions. This paper reports a novel energy-based approach for developing unified models that predict the fatigue life of boiler tube materials in ultra-supercritical (USC) power plants. The proposed method combines the Masing behavior with a cyclic stress–strain relationship and existing stress-based or strain-based fatigue life prediction models. Notably, the developed models conform to the structure of the modified Morrow model, which incorporates material toughness (a temperature compensation parameter) into the Morrow model to account for the effects of temperature. A significant advantage of this approach is that it eliminates the need for tensile tests, which are otherwise essential for assessing material toughness in the modified Morrow model. Instead, all material constants in our models are derived solely from fatigue test results. We validate our models using fatigue data from three promising USC boiler tube materials—Super304H, TP310HCbN, and TP347H—and their welded joints at operating temperatures of 500, 600, and 700 °C. The results demonstrate that approximately 91% of the fatigue data for all six materials fall within a 2.5× scatter band of the model’s predictions, indicating a high level of accuracy and broad applicability across various USC boiler tube materials and their welded joints, which is equivalent to the performance of the modified Morrow model.

## 1. Introduction

The rising concerns over global warming have put traditional coal-fired power plants, which have dominated energy generation for decades, under scrutiny. To address environmental concerns and meet the growing demand for energy, there has been a push towards enhancing efficiency through increased operating temperatures and working fluid pressures. This shift has led to the transition from subcritical (SC) power plants to ultra-supercritical (USC) and, more recently, advanced ultra-supercritical (A-USC) power plants, categorized based on the operational conditions of the working fluid [1,2]. In USC power plants, the working fluid is subjected to elevated temperatures up to 600 °C and pressures as high as 31 MPa. Consequently, key components in USC power plants, like boiler tubes, must be engineered to endure even more severe conditions than those in conventional power plants.

For USC boiler tubes, the selection of materials must ensure two critical properties: creep rupture strength and resistance to steam oxidation [1,2,3]. For instance, it has been reported that low-alloy steels, previously used in the water-cooled wall tubes of SC coal-fired power plants, lack the necessary creep strength for USC applications [4,5]. Hence, significant research efforts have been dedicated to understanding creep and oxidation mechanisms and developing suitable materials [6,7,8,9,10,11,12,13,14,15,16,17,18,19,20,21]. Recent studies have indicated a preference for austenitic steels over conventional ferritic steels for USC applications, with particular emphasis on austenitic steels, like Super304H, TP310HCbN, and TP347H, which demonstrate superior performances at temperatures up to 700 °C [1,2,3].

In addition to creep and oxidation resistance, the fatigue resistance of candidate materials is crucial. The intermittent operation of modern thermal power plants, driven by policy decisions, subjects components like boiler tubes to a significant amount of cyclic thermal stress, particularly at weld connections, which are among the most vulnerable points. However, despite the importance of fatigue properties, there is a relative scarcity of research focusing on the fatigue properties and life prediction models for boiler tube materials in USC power plants [22,23,24,25].

Recent studies have highlighted the modified Morrow model, an energy-based fatigue life prediction approach, for its efficacy in predicting the low-cycle fatigue (LCF) life of austenitic steels used in USC boiler tubes, such as Super304H [23] and TP310HCbN [24]. This model incorporates a temperature compensation parameter (material toughness) into the Morrow model [26,27] to account for the influence of temperature on the fatigue resistance, but it requires tensile testing to determine the material toughness, which is a significant limitation.

In this study, we aim to address the limitations of the modified Morrow model by proposing a novel energy-based approach that allows for the derivation of unified fatigue life prediction models akin to the modified Morrow model. This new method integrates the Masing behavior of the cyclic stress–strain relationship with established models such as the Coffin–Manson [28] or Basquin model [29], eliminating the need for additional tensile tests to determine material constants. We validate the developed models using LCF data from promising USC boiler tube materials—Super304H, TP310HCbN, and TP347H—and their welded joints at operating temperatures of 500, 600, and 700 °C.

## 2. LCF Life Prediction Models and the Proposed Approach

### 2.1. Strain-Based Fatigue Life Prediction Model

When materials are subjected to strain-controlled cyclic loading, they typically reach a stabilized stress–strain response after the initial cyclic hardening or softening, which is influenced by the material’s microstructural characteristics [30]. This stabilized state persists for the majority of the fatigue life. Consequently, the stress–strain hysteresis loop observed at this stabilized cycle accurately reflects the cyclic stress–strain behavior of the material under fatigue deformations.

In LCF deformations, the cyclic stress–strain curve is established by aggregating stress–strain hysteresis loops at various strain amplitudes and linking their apexes. Employing Hooke’s law and the Ramberg–Osgood equation [31], the relationships between the total strain amplitude ($\Delta \varepsilon_{t}/2$), elastic strain amplitude ($\Delta \varepsilon_{e}/2$), plastic strain amplitude ($\Delta \varepsilon_{p}/2$), and stress amplitude (Δσ/2) in LCF deformations can be expressed as follows:

(1) $$\frac{\Delta \varepsilon_{t}}{2}=\frac{\Delta \varepsilon_{e}}{2}+\frac{\Delta \varepsilon_{p}}{2}=\frac{\Delta \sigma }{2E}+{\left(\frac{\Delta \sigma }{2K^{\prime }}\right)}^{1/n^{\prime }}$$

where E, $k^{\prime }$, and $n^{\prime }$ denote the elastic modulus, cyclic strength coefficient, and cyclic strain hardening exponent, respectively.

LCF testing is performed under a strain control with a fixed total strain amplitude, which is subdivided into elastic and plastic components. The Coffin–Manson relation connects the plastic strain amplitude with the fatigue life $N_{f}$ as follows:

(2) $$\left(\frac{\Delta \varepsilon_{p}}{2}\right)=\varepsilon_{f}^{\prime }\cdot {\left(2N_{f}\right)}^{-c}$$

where $\varepsilon_{f}^{\prime }$ and c are the fatigue ductility coefficient and fatigue ductility exponent, respectively. Conversely, the Basquin equation correlates the elastic strain amplitude with the fatigue life as follows:

(3) $$\left(\frac{\Delta \varepsilon_{e}}{2}\right)=\frac{\sigma_{f}^{\prime }}{E}\cdot {\left(2N_{f}\right)}^{-b}$$

where $\sigma_{f}^{\prime }$ and b represent the fatigue strength coefficient and fatigue strength exponent, respectively. By integrating Equations (2) and (3) into Equation (1), a comprehensive relationship between the total strain amplitude and fatigue life can be formulated, as delineated in Equation (4).

(4) $$\left(\frac{\Delta \varepsilon_{t}}{2}\right)=\frac{\sigma_{f}^{\prime }}{E}\;{\left(2N_{f}\right)}^{-b}+\varepsilon_{f}^{\prime }{\left(2N_{f}\right)}^{-c}$$

### 2.2. Energy-Based Fatigue Life Prediction Models

The stress–strain response of materials during fatigue deformations is not always stabilized [30]. Some materials show a continuous change in the stress–strain response with the number of cycles. In this case, the fatigue parameters, such as the elastic strain amplitude and plastic strain amplitude, used in the strain-based fatigue life prediction model are not valid anymore as they continuously vary during fatigue deformations.

A plastic strain energy density, which is defined as the inner area of a stress–strain hysteresis loop, is a combined property of stress and plastic strain. As shown in Equation (1), stress and plastic strain are inversely related to each other under fatigue deformations, making the plastic strain energy density insensitive to the variation in the stress–strain response during fatigue deformations [30]. Therefore, the plastic strain energy density has a desirable feature as a fatigue parameter, which is invariant during fatigue deformations. It is noted that the plastic strain energy density is a composite measure of the fatigue damage at each cycle, as the cyclic plastic strain and stress are related to the movement of dislocations and the resistance against their motion, respectively.

The Morrow model correlates the plastic strain energy density ($\Delta W_{p}$) with the fatigue life as follows:

(5) $$\Delta W_{p}\cdot {\left(2N_{f}\right)}^{m}=C$$

where m and C are the fatigue exponent and material energy absorption capacity, respectively. The Morrow model implies that an identical amount of the plastic strain energy is accumulated for each cycle, and the material will result in a failure if the amount of the accumulated plastic strain energy reaches a critical value, that is, the material’s energy absorption capacity. As the material’s energy absorption capacity varies with temperature, the Morrow model is known to be unable to account for the effect of temperature on the fatigue resistance (i.e., the Morrow model cannot predict the fatigue lives at different temperatures with a single set of material constants).

To overcome this limitation in the Morrow model, a material toughness ($W_{o}$, a temperature compensation parameter) that is defined as the area of the tensile stress–strain curve and represents the material’s capability to absorb strain energy up to the failure has been incorporated into the Morrow model [32]. This is called the modified Morrow model and has the form shown in Equation (6).

(6) $$\frac{\Delta W_{p}}{W_{o}}\cdot {\left(2N_{f}\right)}^{m}=C$$

The applicability of the modified Morrow model has been successfully demonstrated with various stainless steels, including 316L stainless steel [32], 429 EM stainless steel [32], Super304H [23], and TP310HCbN [24]. However, it is noted that there are two major complications in the use of the model. One is the requirement of additional tensile tests to assess the material toughness. The other is the difficulty of calculating the material toughness from the stress–strain curve, which arises from the development of a triaxial stress state after the onset of the necking in the test specimen.

### 2.3. A Proposed Approach for Unified Energy-Based Life Prediction Models

To overcome these complications in the use of the modified Morrow model, we propose a novel method that derives two unified energy-based fatigue life prediction models using a strain- or stress-based approach, which are structurally identical to the modified Morrow model. Unlike the modified Morrow model, however, the developed models do not require additional tensile tests to assess the material toughness. Instead, all materials constants in the models can be derived solely from the fatigue test results.

For materials that exhibit Masing behavior in the cyclic stress–strain relationship (Figure S1), the plastic strain energy density is calculated as given in Equation (7) [33,34]:

(7) $$\Delta W_{p}=\left(\frac{1-n^{\prime }}{1+n^{\prime }}\right)\Delta \sigma \cdot \Delta \varepsilon_{p}$$

Initially, to formulate a unified energy-based model using a strain-based approach, the correlation between Δσ and $\Delta \varepsilon_{p}$ in Equation (1) is integrated into Equation (7) by eliminating Δσ ($=2K^{\prime }\cdot {\left(\frac{\Delta \varepsilon_{p}}{2}\right)}^{n^{\prime }}$), resulting in Equation (8):

(8) $$\Delta W_{p}=\frac{4K^{\prime }\left(1-n^{\prime }\right)}{\left(1+n^{\prime }\right)}\cdot {\left(\frac{\Delta \varepsilon_{p}}{2}\right)}^{1+n^{\prime }}$$

Given that $\Delta \varepsilon_{p}$ is associated with $N_{f}$ through the Coffin–Manson model (Equation (2)), integrating Equation (2) into Equation (8) yields a unified energy-based life prediction model, as shown by Equation (9):

(9) $$\frac{\Delta W_{p}}{4K^{\prime }\cdot {\left(\varepsilon_{f}^{\prime }\right)}^{n^{\prime }+1}}\cdot {\left(2N_{f}\right)}^{c\left(n^{\prime }+1\right)}=\left(\frac{1-n^{\prime }}{1+n^{\prime }}\right)$$

In Equation (9), by setting $4K^{\prime }{\left(\varepsilon_{f}^{\prime }\right)}^{n^{\prime }+1}=W_{o}$, $c\left(n^{\prime }+1\right)=m$ and $\left(\frac{1-n^{\prime }}{1+n^{\prime }}\right)=C$, we obtain a formulation identical to the modified Morrow model (Equation (6)). However, the material constants, $W_{o}$, m, and C, can be directly derived from fatigue test results.

Conversely, to develop an alternative unified energy-based model, we apply a stress-based approach. The plastic strain energy density in Equation (7) is alternatively expressed by eliminating $\Delta \varepsilon_{p}$. Based on Equation (1), where $\Delta \varepsilon_{p}=2\cdot {\left(\frac{\Delta \sigma }{2K^{\prime }}\right)}^{\frac{1}{n^{\prime }}}$, integrating this relationship into Equation (7) leads to Equation (10):

(10) $$\Delta W_{p}=\frac{4\left(1-n^{\prime }\right)}{\left(1+n^{\prime }\right){\left(K^{\prime }\right)}^{1/n^{\prime }}}\cdot {\left(\frac{\Delta \sigma }{2}\right)}^{\frac{n^{\prime }+1}{n^{\prime }}}$$

According to the Basquin model (Equation (3)), where Δσ is linked to $N_{f}$, integrating Equation (3) into Equation (10) establishes the relationship between $\Delta W_{p}$ and $N_{f}$, as shown in Equation (11):

(11) $$\frac{\Delta W_{p}}{4{\left(K^{\prime }\right)}^{\left(-\frac{1}{n^{\prime }}\right)}\cdot {\left(\sigma_{f}^{\prime }\right)}^{\left(\frac{n^{\prime }+1}{n^{\prime }}\right)}}\cdot {\left(2N_{f}\right)}^{b\left(\frac{n^{\prime }+1}{n^{\prime }}\right)}=\left(\frac{1-n^{\prime }}{1+n^{\prime }}\right)$$

By setting $4{\left(K^{\prime }\right)}^{\left(-\frac{1}{n^{\prime }}\right)}\cdot {\left(\sigma_{f}^{\prime }\right)}^{\left(\frac{n^{\prime }+1}{n^{\prime }}\right)}=W_{o}$, $b\cdot \left(\frac{n^{\prime }+1}{n^{\prime }}\right)=m$, and $\left(\frac{1-n^{\prime }}{1+n^{\prime }}\right)=C$, Equation (11) adopts the same structural format as the modified Morrow model. Similar to Equation (9), the material constants $W_{o}$, m, and C in the model can be exclusively derived from fatigue test results.

## 3. Test Methods for Validating Fatigue Life Prediction Models

### 3.1. Materials and Test Specimen Preparation

To examine the validity of the developed unified energy-based fatigue life prediction models, three promising candidates for USC boiler tube materials, including Super304H, TP310HCbN, and TP347H, and their welded joints were used. The three base metals (BMs) are all austenitic stainless steels. The chemical composition of the three base metals and three filler metals for the welded joints is given in Table 1.

Tensile and LCF test specimens for base metals were machined from base metal tubes with a wall thickness of 7.0 mm. The outer diameter was 42.5 mm for Super304H tubes, 44.5 mm for TP310HCbN tubes, and 38.0 mm for TP347H tubes. The tensile test specimens had a gauge section of 30 mm in length and 6 mm in diameter, whereas the fatigue test specimens had a gauge section of 16 mm in length and 6 mm in diameter. The longitudinal direction of both the tensile and LCF specimens was aligned with the tube axis.

To prepare welded tubes, base metal tubes were cut into smaller tubes with lengths of 50 mm and 70 mm and then welded using a total of five passes of direct current electrode negative (straight polarity) gas tungsten arc welding (GTAW), where a 60° V-shaped groove and a 3 mm root gap were created on each side of the base metal tubes. The filler metals for the welding of Super304H, TP310HCbN, and TP347H tubes were T304-H, ER310, and ER347, respectively (Table 1).

The welding parameters were carefully selected in consideration of practical USC boiler tube constructions. The welding voltage and current were 13 V and 130 A, respectively, and the welding speed was approximately 10 cm/min, operating manually. Following the GTAW process, the welding integrity was examined by conducting penetration tests and radiographic tests.

The tensile and LCF test specimens for welded joints had the same dimensions as those of the base metal specimens, where the specimen axis was aligned perpendicular to the weld axis. In both the specimens, the gauge section included the three welding zones, i.e., the weld metal, heat-affected zone, and base metal, where the weld metal was located at the center. More details on the specimen preparation can be found in our previous works [23,24,25].

### 3.2. Tensile and Low-Cycle Fatigue Tests

Tensile and low-cycle fatigue tests were conducted using hydraulic servo-controlled testing machine (Landmark, MTS, Eden Prairie, MN, USA) with a 100 kN capacity in a temperature range of 23 to 750 °C. The test temperature was maintained within a variation of ±1 °C using a three-zone resistance type furnace (653, MTS, USA). A high-temperature uniaxial extensometer with a gauge length of 12 mm (632.53F-14, MTS, USA) was employed to measure and control the strain in the gauge section of the test specimen.

Tensile tests were carried out at eleven temperatures of 23, 100, 200, 300, 400, 500, 550, 600, 650, 700, and 750 °C, following ISO 6892-1 and 6892-2 standards [35,36]. The test specimens were deformed initially at a constant strain rate of 2.5 × 10^−4^ s^−1^ up to 1% using the high-temperature extensometer, and then the control mode was changed to a displacement-controlled test with a constant rate of 3 mm/min. until the final fracture. For the three base metals, tensile tests were conducted three times at all test temperatures. In contrast, for the three welded joints, they were tested once at temperatures below 300 °C and twice at temperatures above 400 °C.

Low-cycle fatigue tests were conducted in strain control mode using the high-temperature extensometer at test temperatures of 500, 600, and 700 °C, following the ASTM E606 standard [37]. A fully-reversed triangular strain waveform was applied to the specimens at a constant strain rate of 5 × 10^−3^ s^−1^. The total strain amplitudes ranged from 0.2% to 1.0%. For all conditions, each test was performed three times for the base metals and twice for the welded joints.

## 4. Results and Discussion

### 4.1. Tensile and Cyclic Stress–Strain Responses

#### 4.1.1. Tensile Stress–Strain Behavior and Properties

**Figure 1:**
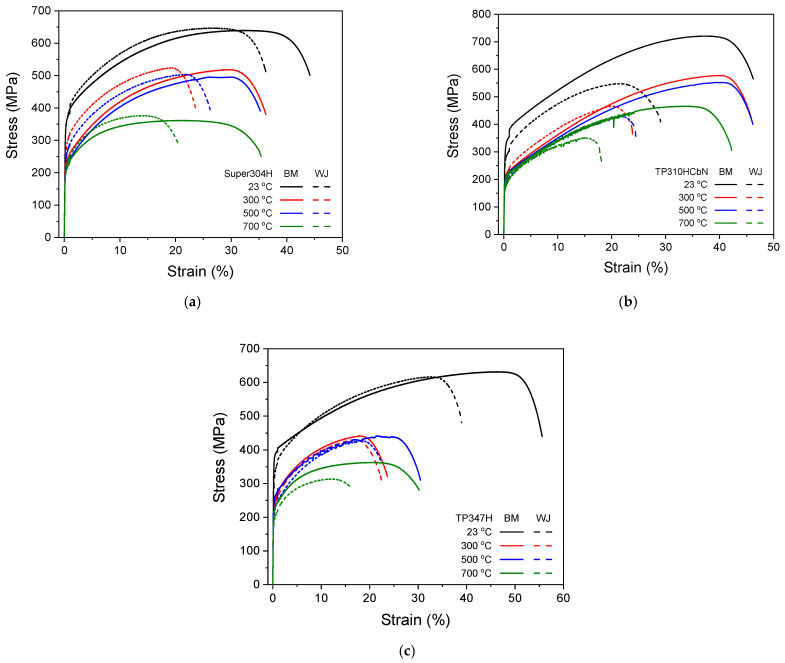
Tensile stress–strain curves of three base metals and their welded joints at temperatures of 23, 300, 500, and 700 °C. (**a**) Super304H, (**b**) TP310HCbN, and (**c**) TP347H.

**Figure 2:**
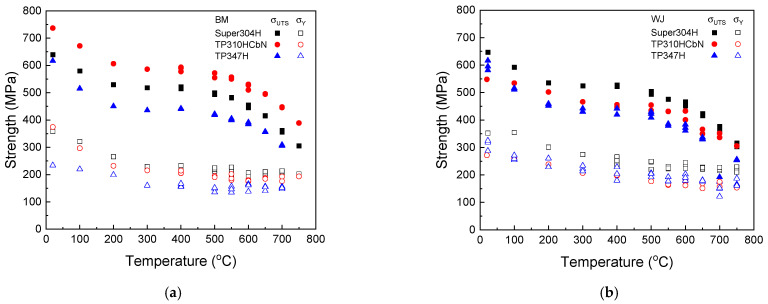

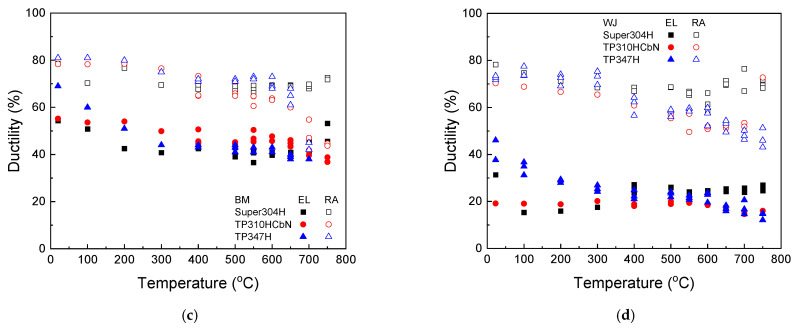
Variation in strength and ductility with temperature. Tensile and yield strengths of (**a**) three base metals and (**b**) their welded joints. Elongation and reduction of area of (**c**) three base metals and (**d**) their welded joints.

Figure 1 presents the tensile stress–strain curves of three base metals (Super304H, TP310HCbN, and TP347H) and their welded joints at four representative temperatures of 23, 300, 500, and 700 °C. Their tensile properties, including their yielding strength, tensile strength, elongation, and reduction of area, obtained from the tensile test results, are shown in Figure 2. For all of the base metals and welded joints, there was a gradual decrease in strength with increasing temperature. However, the decrease was not significant up to 700 °C compared to the values at lower temperatures (Figure 2a,b). As for the ductility, both the elongation and reduction of area values remained nearly constant without any notable decrease, even at high temperatures (Figure 2c,d). These results demonstrate the superior mechanical properties of heat-resistant austenitic steels at high temperatures.

Upon examining the variation in the tensile strength with temperature (Figure 2a,b), it was observed that for all of the materials, the decrease in tensile strength was slowed or a slight increase was noted in the temperature range from 250 to 600 °C. This phenomenon is known to arise from material embrittlement caused by dynamic strain aging, which results from the interaction between moving dislocations and diffusing solute atoms during plastic deformations [38,39,40,41].

When comparing the tensile strength of the base metals and their welded joints (Figure 2a,b), Super304H and TP347H exhibited comparable values across all temperatures for both the base metal and the welded joint, indicating that the welding process does not significantly reduce the strength of the materials. However, for TP310HCbN, the welded joint showed a considerably lower strength compared to that of the base metal, signifying a significant reduction in material strength due to the welding process.

In terms of ductility (Figure 2c,d), regarding the reduction of area, both the base metals and welded joints presented nearly identical values at all temperatures, suggesting that the welding process does not adversely affect the reduction of area in the base metals. Nevertheless, the elongation of the welded joints was notably lower than that of the base metals. This is attributed to the earlier onset of necking in the welded joints compared to the base metals. Our previous studies indicate that inhomogeneities in the material within the welding zone lead to local variations in material properties, causing localized deformation in the softest regions of the welded joint (e.g., the weld metal in the Super304H [23,25] and TP310HCbN [24] welded joints), which in turn results in premature necking.

**Figure 3:**
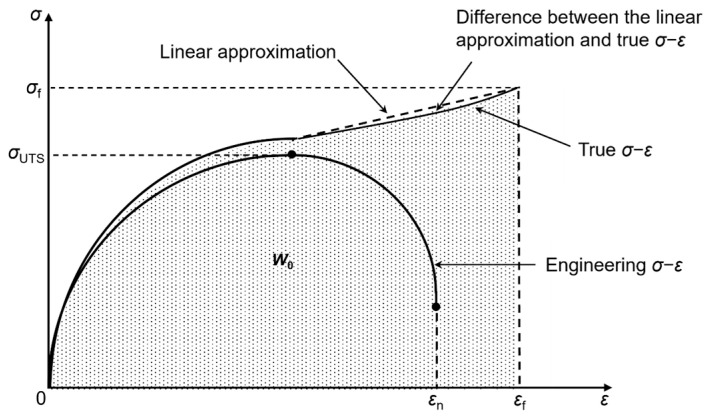
Schematic of calculating material toughness (*W_o_*) from the tensile stress–strain curve using the linear approximation method.

As described in Section 2.2, for the application of the modified Morrow model, Equation (6), to fatigue life predictions, it is necessary to assess the material toughness from tensile test results. Figure 3 illustrates the method for assessing a material’s toughness from the tensile stress–strain curve. In the case of ductile metallic materials, specimen deformations localize at the onset of necking, leading to a triaxial stress state. This condition complicates the determination of an accurate stress–strain relationship and, consequently, the assessment of material toughness. To overcome this challenge, a linear approximation method has been proposed and validated across various metallic materials [32]. This method straightforwardly connects two points on the true stress–strain curve: the necking point and the final fracture point. The material toughness values, calculated using the linear approximation method, for the three base metals and their welded joints are given in Table 2.

#### 4.1.2. Cyclic Stress–Strain Behavior

As outlined in Section 2, the majority of fatigue life prediction models are based on the premise that the fatigue damage parameter value remains constant throughout the entire fatigue life, indicating that an equal amount of fatigue damage accumulates in each cycle. In this context, it is crucial to understand the cyclic stress–strain behavior and its stabilization during fatigue deformations and, further, to identify a suitable fatigue damage parameter for developing an accurate fatigue life prediction model.

**Figure 4:**
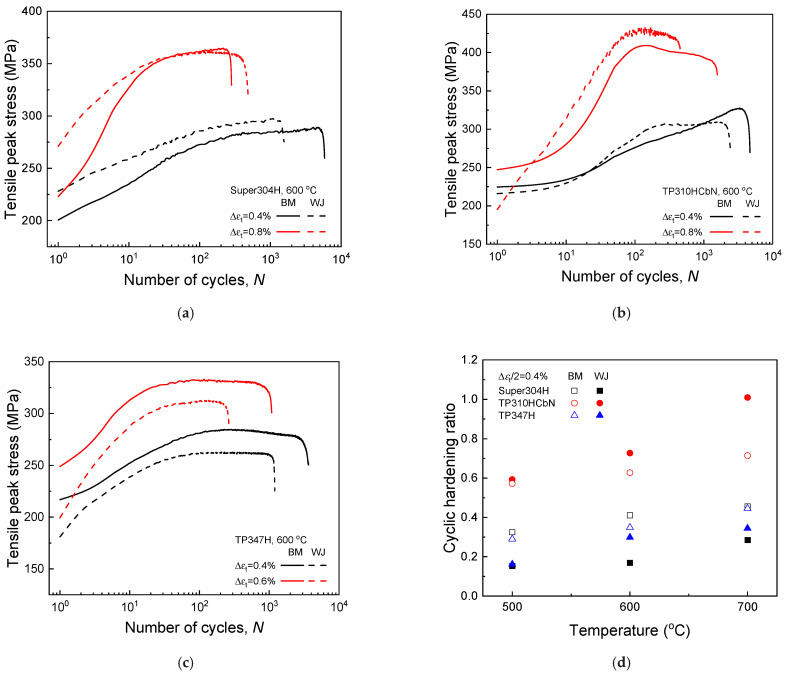
Variation in tensile peak stress with the number of cycles during fatigue deformation. (**a**) Super304H and its welded joint (Δε_t_/2 = 0.4 and 0.8% at 600 °C), (**b**) TP310HCbN and its welded joint (Δε_t_/2 = 0.4 and 0.8% at 600 °C), (**c**) TP347H and its welded joint (Δε_t_/2 = 0.4 and 0.6% at 600 °C), and (**d**) cyclic hardening ratio at temperatures of 500, 600, and 700 °C (Δε_t_/2 = 0.4%).

Figure 4 illustrates how the tensile peak stress changes with the number of cycles (i.e., the cyclic stress response) for the three base metals and their welded joints during a fatigue deformation at 600 °C. All of the base metals and welded joints showed significant cyclic hardening, which is characteristic of annealed metallic materials [24,30]. The cyclic stress response progresses through three stages, as depicted in Figure 4a–c. Initially, the tensile peak stress quickly rises to a maximum within approximately 100 cycles (Stage I—the cyclic hardening region), then remains mostly constant for the majority of the fatigue life (Stage II—the stabilized region). Ultimately, it sharply declines, leading to the final fracture (Stage III—the main crack growth region).

To quantitatively analyze the cyclic hardening behavior, we introduce the cyclic hardening ratio. This ratio measures the increase in tensile peak stress from the first cycle to the midpoint of the fatigue life (i.e., half-life) as shown below:

(12) $$\mathrm{Cyclic}\;\mathrm{hardening}\;\mathrm{ratio}=\frac{\sigma_{TP|N_{f}/2}-\sigma_{TP|1^{st}}}{\sigma_{TP|1^{st}}}$$

where $\sigma_{TP|1^{st}}$ and $\sigma_{TP|N_{f}/2}$ represent the tensile peak stresses at the first cycle and half-life, respectively. Figure 4d displays the cyclic hardening ratio values at Δ*ε*_t_/2 = 0.4% for temperatures of 500, 600, and 700 °C. Across all of the base metals and welded joints, the cyclic hardening ratio increases with the temperature. It also rises with increasing strain amplitude at any given temperature. The underlying mechanisms for the temperature and strain amplitude dependence of cyclic hardening behavior are discussed in our previous study [24]. The TP310HCbN base metal and welded joint showed the highest values, indicating the most pronounced cyclic hardening during the fatigue deformation. Notably, the cyclic hardening ratio of approximately 1 at 700 °C for the welded joint suggests that the tensile peak stress at the half-life is double that at the first cycle. These findings highlight that all of the base metals and their welded joints experience significant changes in their stress–strain relationships during fatigue deformations, underscoring the importance of the careful selection of a fatigue damage parameter when developing a fatigue life prediction model.

### 4.2. Fatigue Damage Parameters

**Figure 5:**
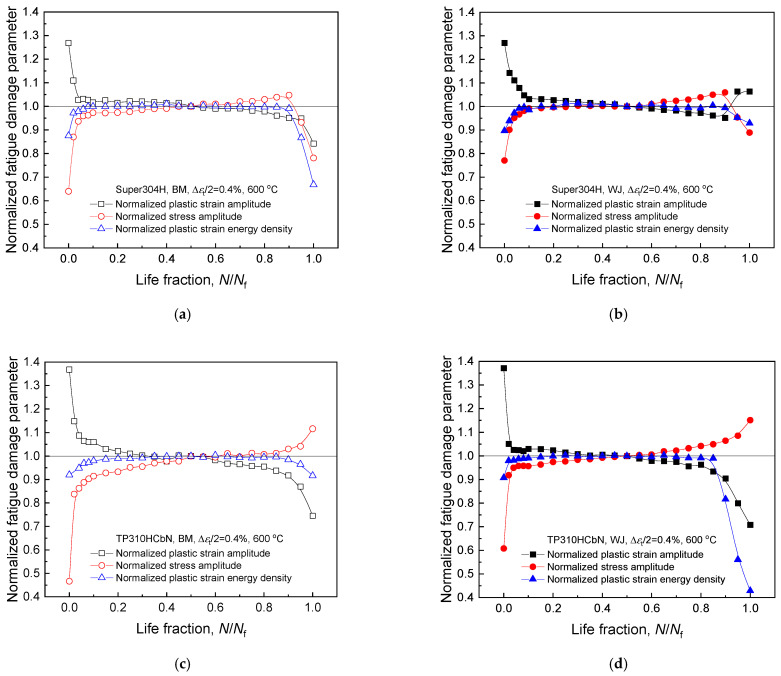

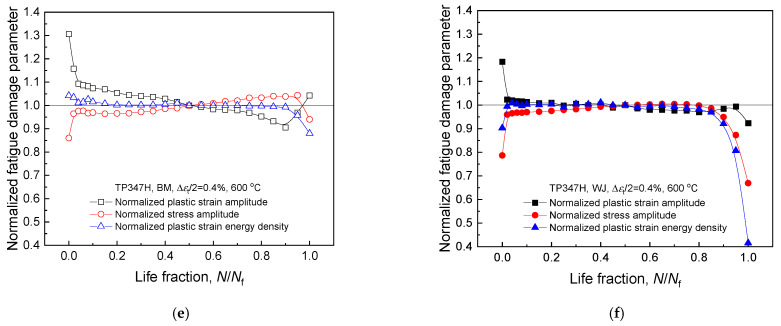
Variation in three fatigue damage parameters with the number of cycles during fatigue deformations (Δ*ε*_t_/2 = 0.4% at 600 °C). (**a**) Super304H and (**b**) its welded joint. (**c**) TP310HCbN and (**d**) its welded joint. (**e**) TP347H and (**f**) its welded joint. Each fatigue damage parameter is normalized by the value at the half-life.

To identify an appropriate fatigue damage parameter that must remain constant during fatigue deformations, we examined three major fatigue damage parameters: the plastic strain amplitude, stress amplitude, and plastic strain energy density. Figure 5 illustrates the variations in these three fatigue damage parameters with the number of cycles under a specific test condition (Δ*ε*_t_/2 = 0.4% at 600 °C), where the value of each fatigue damage parameter is normalized to its value at the half-life. For all of the base metals and welded joints, the plastic strain amplitude and stress amplitude varied continuously with the number of cycles and did not reach a stabilized state due to the significant cyclic hardening of the materials. This reveals that neither the plastic strain amplitude nor the stress amplitude fulfill the criteria for a fatigue damage parameter, rendering them unsuitable for use in the development of a fatigue life prediction model.

In contrast, the plastic strain energy density stabilized within about 10% of the fatigue life, and this stable condition was maintained for the majority of the fatigue life (up to approximately 90%, corresponding to the beginning of stage III in the cyclic stress response curve). This demonstrates the desirable characteristics of the fatigue damage parameter. Notably, consistent results were observed for all of the materials under various test conditions of different strain amplitudes and temperatures. Consequently, the plastic strain energy density is deemed an appropriate fatigue damage parameter for the development of a fatigue life prediction model for the USC boiler tube materials investigated in this study. The physical meaning of the plastic strain energy density and its invariance during fatigue deformations, despite considerable cyclic hardening, are discussed in Section 2.2.

### 4.3. Comparison of Fatigue Life Prediction Models and Verification of the Proposed Approach

**Figure 6:**
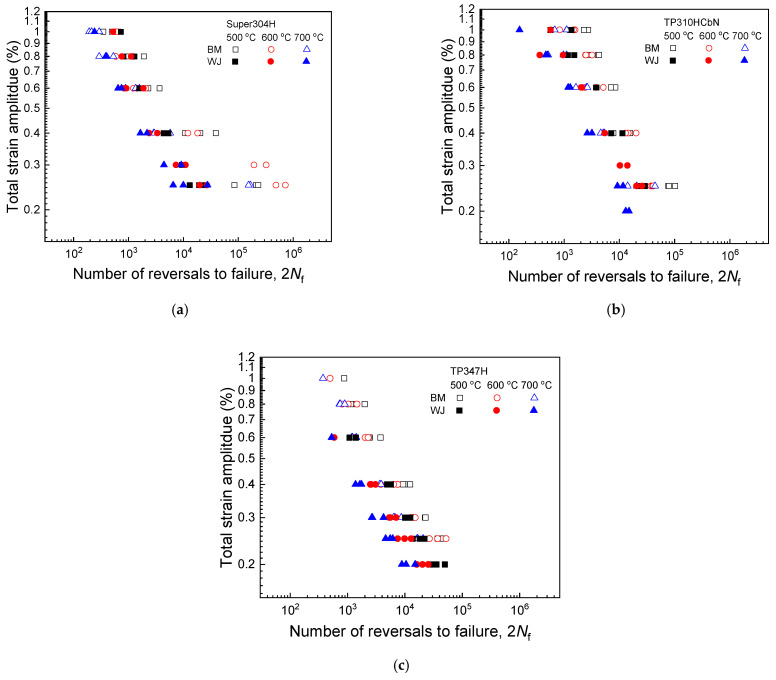
Total strain amplitude versus fatigue life data at temperatures of 500, 600, and 700 °C. (**a**) Super304H and its welded joint, (**b**) TP310HCbN and its welded joint, and (**c**) TP347H and its welded joint.

Figure 6 presents the total strain amplitude–fatigue life data for the three base metals and their welded joints at temperatures of 500, 600, and 700 °C. For all materials, the fatigue life (i.e., fatigue resistance) decreased with increasing strain amplitude and temperature, exhibiting the typical fatigue resistance characteristics of metallic materials. Additionally, the welded joints showed a lower level of fatigue resistance compared to that of their counterpart base metals, indicating the adverse effect of the welding process on the fatigue resistance. The underlying mechanisms for this degradation in fatigue resistance of the welded joints related to microstructural aspects have been discussed in our previous studies [23,24,25].

**Figure 7:**
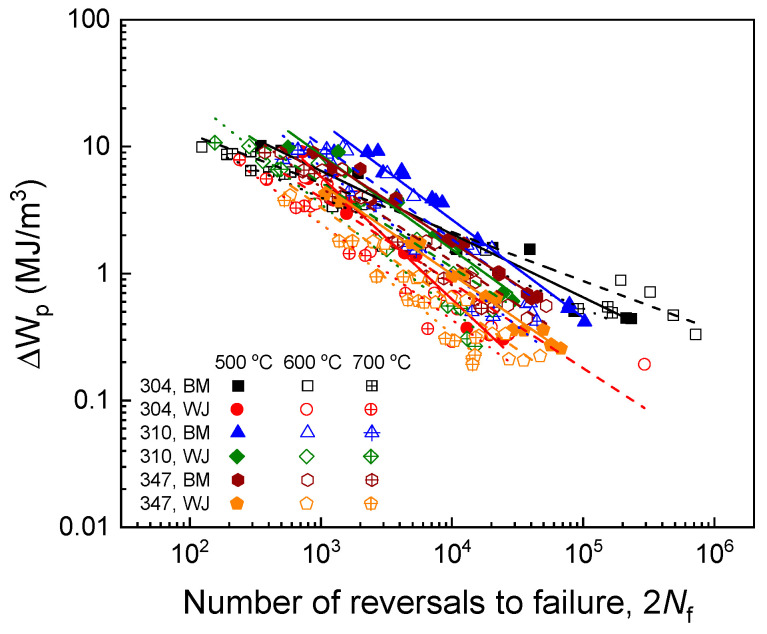
Fatigue life predictions for three base metals and their welded joints using the Morrow model shown in Equation (5).

As mentioned in Section 4.2, the plastic strain energy density was identified as a suitable fatigue damage parameter for all six of the materials. Consequently, fatigue life prediction models for USC boiler tube materials were developed based on the plastic strain energy density. Initially, we evaluated the Morrow model, as shown in Equation (5). As depicted in Figure 7, a linear correlation exists between the plastic strain energy density and the fatigue life under isothermal conditions for all of the materials, confirming the model’s robust predictive capability. The material constants used in the model predictions are listed in Table 2. However, it was observed that the data at the three different temperatures did not align on a single line, indicating the model’s inability to capture the effect of temperature on fatigue resistance.

Upon examining the variation in the Morrow model’s material constants, m and C, with temperature (Table 3), it was found that the m value remained almost unchanged, whereas the C value varied significantly. Given the physical meaning of the material constant C, this variation with the temperature suggests that the material’s energy absorption capability is substantially influenced by temperature, thus predominating over its fatigue resistance.

**Figure 8:**
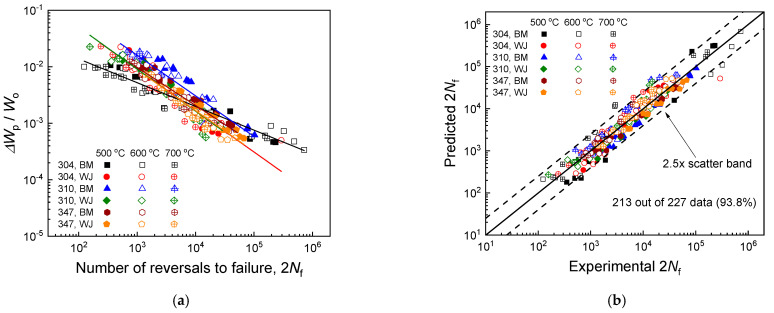
Fatigue life predictions for three base metals and their welded joints using the modified Morrow model shown in Equation (6). (**a**) Predictions made by the modified Morrow model. (**b**) Comparison of experimental results with model predictions.

To accommodate the change in a material’s energy absorption capability with temperature and the consequent effects on its fatigue resistance, material toughness was introduced as a temperature compensation parameter in the Morrow model, leading to the formulation of the modified Morrow model, as shown in Equation (6). Figure 8 showcases the fatigue life predictions for three base metals and their welded joints using the modified Morrow model. The material constants (m, C) used in the model predictions were calculated to be (0.42, 0.10) for the Super304H base metal, (0.73, 1.45) for the Super304H welded joint, (0.72, 2.32) for the TP310HCbN base metal, (0.73, 1.33) for the TP310HCbN welded joint, (0.67, 1.03) for the TP347H base metal, and (0.72, 1.30) for the TP347H welded joint. In calculating (m, C), the material toughness values from Table 2 were employed. For all six materials, the data at three different temperatures aligned on a single line, illustrating the model’s high prediction accuracy (Figure 8a). To quantitatively evaluate the model’s prediction accuracy, the proportion of experimental data falling within the 2.5× scatter band of the model’s predictions to the total experimental data was calculated. As indicated in Figure 8b, 213 out of 227 data points (~94%) fell within the 2.5× scatter band of the model’s predictions, validating the model’s extensive applicability across various USC boiler tube materials and its prediction accuracy.

**Figure 9:**
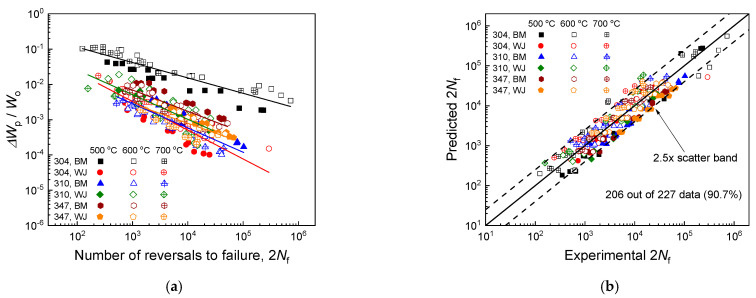
Fatigue life predictions for three base metals and their welded joints using the proposed unified energy-based model developed via the strain-based approach shown in Equation (9). (**a**) Predictions made by the proposed model. (**b**) Comparison of experimental results with model predictions.

Figure 9 presents the fatigue life predictions made by the proposed unified energy-based model developed through the strain-based approach shown in Equation (9). The material constants (m, C, $W_{o}$) used in the model predictions were calculated to be (0.43, 0.69, 138) for the Super304H base metal, (0.80, 0.72, 1570) for the Super304H welded joint, (0.79, 0.69, 3310) for the TP310HCbN base metal, (0.73, 0.70, 1126) for the TP310HCbN welded joint, (0.67, 0.70, 783) for the TP347H base metal, and (0.75, 0.72, 785) for the TP347H welded joint. As outlined in Section 2.3, the material constants were derived from the fatigue data at each temperature, and the averages over the three temperatures were taken for the model predictions (Tables S1 and S2). The results demonstrated that for all six materials, the fatigue life was linearly correlated with $\Delta W_{p}/W_{o}$ on a log–log scale, consolidating all of the data from three different temperatures into a single line (Figure 9a). This affirmed the outstanding prediction accuracy of the proposed model (Figure 9a). The comparison of the model predictions with the experimental results revealed that 206 out of 227 data points (~91%) fell within the 2.5× scatter band of the model predictions, confirming the model’s high prediction accuracy and its broad applicability across various materials (Figure 9b).

**Figure 10:**
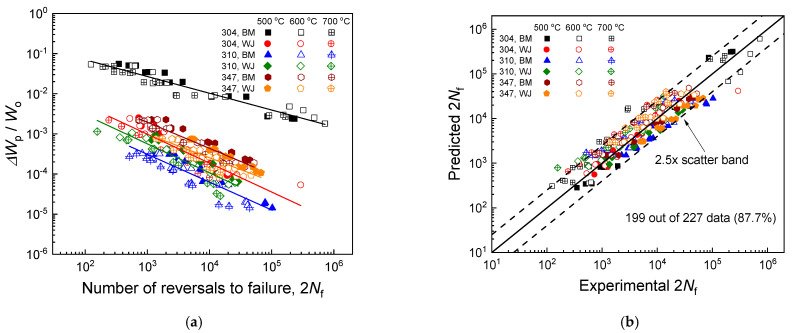
Fatigue life predictions for three base metals and their welded joints using the proposed unified energy-based model developed via the stress-based approach shown in Equation (11). (**a**) Predictions made by the proposed model. (**b**) Comparison of experimental results with model predictions.

The fatigue life predictions made by the proposed unified energy-based model developed via the stress-based approach shown in Equation (11) are illustrated in Figure 10. The material constants (m, C, $W_{o}$) used in the model predictions were calculated to be (0.45, 0.69, 184) for the Super304H base metal, (0.89, 0.72, 3580) for the Super304H welded joint, (1.05, 0.69, 29232) for the TP310HCbN base metal, (0.96, 0.70, 9413) for the TP310HCbN welded joint, (0.78, 0.70, 2934) for the TP347H base metal, and (0.86, 0.72, 2385) for the TP347H welded joint. These material constants were obtained following the same methodology used in Equation (9) (Tables S1 and S3). The findings showed that for all six materials, a linear relationship existed between the fatigue life and $\Delta W_{p}/W_{o}$ on a log–log scale (Figure 10a). As depicted in Figure 10b, 199 out of 227 data points (~88%) fell within the 2.5× scatter band of the model’s predictions, evidencing the model’s reliable prediction accuracy and its widespread applicability across different materials.

Based on these findings, we can conclude that the proposed unified energy-based models shown in Equations (9) and (11) demonstrate equivalent levels of prediction accuracy and applicability compared to those of the modified Morrow model. Furthermore, they overcome the limitation of the modified Morrow model, which necessitates tensile testing to evaluate material toughness and to thereby account for the effects of temperature on fatigue resistance.

## 5. Conclusions

In this study, we developed unified energy-based fatigue life prediction models by integrating the Masing behavior observed in cyclic stress–strain relationships with established strain-based (Coffin–Manson relation) or stress-based (Basquin relation) models. The developed models are structurally identical to the modified Morrow model, which incorporates a temperature compensation parameter (material toughness) to address the effects of temperature on fatigue resistance. Unlike the modified Morrow model, however, our models do not require additional tensile testing to determine the material toughness; instead, all of the necessary material constants were derived from the fatigue test results. We validated the efficacy of these models using three promising USC boiler tube materials (Super304H, TP310HCbN, and TP347H) and their welded joints. The results showed that for all of the base metals and their welded joints, approximately 91% of the experimental data fell within a 2.5× scatter band of the models’ predictions, demonstrating their high prediction accuracy and extensive applicability across various materials. This performance is comparable to that of the modified Morrow model.

## Figures and Tables

**Table 1:** Chemical composition of the three base metals and filler metals (wt%).

Material	C	Si	Mn	Cr	Ni	Mo	N	Nb	Cu	P	S	Fe
Super304H (BM)	0.09	0.21	0.76	17.8	8.39	-	0.2	0.42	2.87	0.06	0.01	Bal.
T-304H (Filler)	0.13	0.3	3.2	18.0	16.0	0.9	0.15	0.60	3.0	0.03	0.03	Bal
TP310HCbN (BM)	0.07	0.39	1.13	24.6	19.5	-	0.47	0.39	-	0.03	0.01	Bal.
ER310 (Filler)	0.01	0.50	1.70	26.0	21.0	0.15	-	-	0.75	0.01	0.01	Bal
TP347H (BM)	0.05	0.51	1.39	16.4	9.22	-	-	0.43	-	0.03	0.01	Bal.
ER347 (Filler)	0.06	0.45	1.76	20.3	9.98	0.11	-	0.39	0.21	0.03	0.01	Bal.

**Table 2:** Material toughness $W_{o}$ values of three base metals and their welded joints at temperatures ranging from 23 to 700 °C (unit: MJ/m^3^).

Temperature (°C)		23	100	200	300	400	500	550	600	650	700
Super304H	BM	2098.8 (-)	1736.1 (-)	1541.2 (-)	1415.5 (-)	1209.0 (94.1) ^a^	1050.5 (38.6)	955.5 (53.0)	984.9 (82.7)	934.8 (34.9)	905.7 (21.6)
	WJ	997.6 (-)	915.5 (-)	790.8 (-)	714.6 (-)	599.4 (6.9)	462.1 (5.0)	409.6 (90.7)	385.0 (11.1)	360.4 (14.4)	340.7 (30.7)
TP310HCbN	BM	1325.5 (9.6)	1135.9 (4.4)	1043 (20.5)	917.6 (11.8)	806.6 (9.4)	788.6 (12.3)	670.0 (20.9)	593.9 (13.3)	551.0 (6.8)	516.0 (12.3)
	WJ	1135.9 (-)	945.3 (-)	915.6 (-)	854.6 (-)	788.6 (22.6)	754.3 (18.5)	656.4 (12.5)	615.7 (41.5)	521.5 (6.5)	475.8 (18.2)
TP347H	BM	1744.5 (23.5)	1431.5 (19.9)	1141.5 (27.5)	952.9 (34.1)	764.8 (27.2)	691.1 (15.6)	662.3 (32.2)	615.1 (41.5)	574.3 (13.7)	533.3 (17.1)
	WJ	1318.5 (25.1)	1326.6 (29.5)	904.61 (31.2)	919.9 (30.2)	548.0 (14.5)	472.8 (10.6)	450.8 (23.9)	407.2 (24.6)	324.1 (11.5)	245.8 (23.0)

^a^ The number in parentheses indicates the standard deviation.

**Table 3:** Material constants for the Morrow model of three base metals and their welded joints at temperatures of 500, 600, and 700 °C.

	BM						WJ					
Temp. (°C)	500		600		700		500		600		700	
Material Constant	*m*	*C* (MJ/m^3^)	*m*	*C* (MJ/m^3^)	*m*	*C* (MJ/m^3^)	*m*	*C* (MJ/m^3^)	*m*	*C* (MJ/m^3^)	*m*	*C* (MJ/m^3^)
Super304H	0.5	202	0.39	75	0.44	82	0.97	4667	0.67	412	0.76	453
TP310HCbN	0.77	3232	0.74	1739	0.84	2314	0.75	1493	0.72	807	0.84	1136
TP347H	0.67	873	0.68	668	0.77	1004	0.71	694	0.75	580	0.88	1055

## Data Availability

All data and methods are presented in the main text and the Supplementary Materials. All other relevant data are available from the corresponding authors upon reasonable request.

## Supplementary Materials

The following supporting information can be downloaded at: https://www.mdpi.com/article/10.3390/ma17102186/s1, Table S1: Material constants for the Coffin–Manson (Equation (2)) and Basquin (Equation (3)) models of three base metals and their welded joints; Table S2: Material constants for the proposed unified energy-based model, Equation (9), developed using the strain-based approach, for three base metals and their welded joints; Table S3: Material constants for the proposed unified energy-based model, Equation (12), developed using the stress-based approach, for three base metals and their welded joints; Figure S1: Masing behavior of stabilized stress–strain hysteresis loops at half-life for three base metals and their welded joints.
