# Supplementary material for: Energy-Based Unified Models for Predicting the Fatigue Life Behaviors of Austenitic Steels and Welded Joints in Ultra-Supercritical Power Plants
Source: Materials (Basel). 2024 May 7;17(10):2186. doi: 10.3390/ma17102186 (PMC11123133; doi:10.3390/ma17102186)
Supplement: Supplementary file 1 [file materials-17-02186-s001.zip › materials-2979237-supplementary.pdf]

Supplementary Materials for

Energy-Based Unified Models for Predicting the Fatigue Life Behaviors of Austenitic Steels and Welded Joints in Ultra-Supercritical Power Plants

Jeong Ho Hwang [1,†], Dae-Woong Kim [2,3,4,†], Jae-Yong Lim [4,*] and Seong-Gu Hong [2,3,5,*]

1 Institute of Future Energy Technology, FNC Technology Co., Ltd., Yongin 17084, Republic of Korea; jhhwang@fnctech.com
2 Convergence Research Center for Meta-Touch, Korea Research Institute of Standards and Science, Daejeon 34113, Republic of Korea; kimdw@kriss.re.kr
3 Division of Chemical and Material Metrology, Korea Research Institute of Standards and Science, Daejeon 34113, Republic of Korea
4 Department of Safety Engineering, Seoul National University of Science and Technology, Seoul 01811, Republic of Korea
5 Department of Nano Science, University of Science and Technology, Daejeon 34113, Republic of Korea
* Correspondence: jaeyong.lim@gmail.com (J.-Y.L.); sghong@kriss.re.kr (S.-G.H.); Tel.: +82-2-970-6388 (J.-Y.L.); +82-42-868-5868 (S.-G.H.)
† These authors contributed equally to this work.

Table S1. Material constants for the Coffin-Manson (Eq. (2)) and Basquin (Eq. (3)) models of three base metals and their welded joints. 304, 310, and 347 correspond to Super304H, TP310HCbN, and TP347H, respectively.

	500 ℃					600 ℃					700 ℃				
	ε_f'	c	$\frac{\sigma_f'}{E}$	b	E (GPa)	ε_f'	c	$\frac{\sigma_f'}{E}$	b	E (GPa)	ε_f'	c	$\frac{\sigma_f'}{E}$	b	E (GPa)
304, BM	0.083	0.396	4.68×10^{-3}	0.094	165.5	0.047	0.337	3.31×10^{-3}	0.056	155.4	0.044	0.351	3.36×10^{-3}	0.059	141.0
304, WJ	0.837	0.752	6.87×10^{-3}	0.143	146.0	0.403	0.670	6.55×10^{-3}	0.134	139.7	0.205	0.640	4.17×10^{-3}	0.099	138.2
310, BM	0.706	0.611	8.12×10^{-3}	0.144	160.0	0.944	0.677	9.71×10^{-3}	0.170	160.0	0.703	0.716	8.98×10^{-3}	0.163	150.0
310, WJ	0.369	0.583	8.33×10^{-3}	0.167	166.7	0.192	0.546	6.27×10^{-3}	0.132	161.3	0.563	0.725	5.37×10^{-3}	0.133	150.9
347, BM	0.176	0.482	8.31×10^{-3}	0.160	162.0	0.262	0.565	4.71×10^{-3}	0.102	160.0	0.472	0.674	3.79×10^{-3}	0.088	155.0
347, WJ	0.319	0.592	4.34×10^{-3}	0.113	158.1	0.239	0.612	5.14×10^{-3}	0.138	151.5	0.433	0.732	4.14×10^{-3}	0.113	150.1

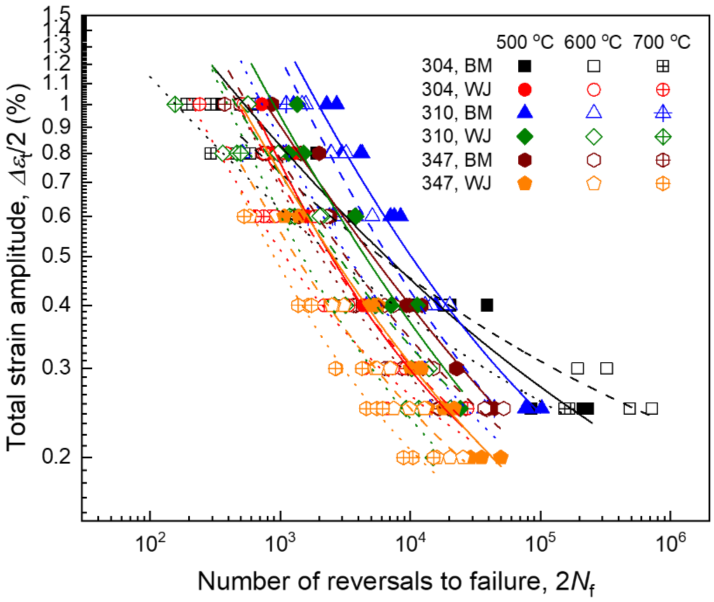

Table S2. Material constants for the proposed unified energy-based model, Eq. (9), developed using the strain-based approach, for three base metals and their welded joints.

	500 °C			600 °C			700 °C		
	W_o	m	C	W_o	m	C	W_o	m	C
Super304H, BM	238	0.49	0.62	96	0.39	0.73	80	0.41	0.73
Super304H, WJ	2994	0.88	0.71	1270	0.79	0.71	446	0.73	0.75
TP310HCbN, BM	2451	0.71	0.71	4746	0.83	0.64	2732	0.83	0.73
TP310HCbN, WJ	1433	0.71	0.63	532	0.64	0.71	1414	0.83	0.75
TP347H, BM	613	0.61	0.59	705	0.66	0.71	1031	0.75	0.80
TP347H, WJ	829	0.70	0.69	567	0.72	0.71	960	0.84	0.75

Table S3. Material constants for the proposed unified energy-based model, Eq. (12), developed using the stress-based approach, for three base metals and their welded joints.

	500 °C			600 °C			700 °C		
	W_o	m	C	W_o	m	C	W_o	m	C
Super304H, BM	353	0.49	0.62	97	0.42	0.73	102	0.43	0.73
Super304H, WJ	6521	0.97	0.71	3511	0.91	0.71	707	0.80	0.75
TP310HCbN, BM	41213	1.01	0.71	15273	0.93	0.64	31211	1.22	0.73
TP310HCbN, WJ	10101	0.91	0.63	6927	0.90	0.71	11212	1.08	0.75
TP347H, BM	5185	0.78	0.59	1495	0.69	0.71	2123	0.87	0.80
TP347H, WJ	1180	0.74	0.69	3613	0.94	0.71	2361	0.89	0.75

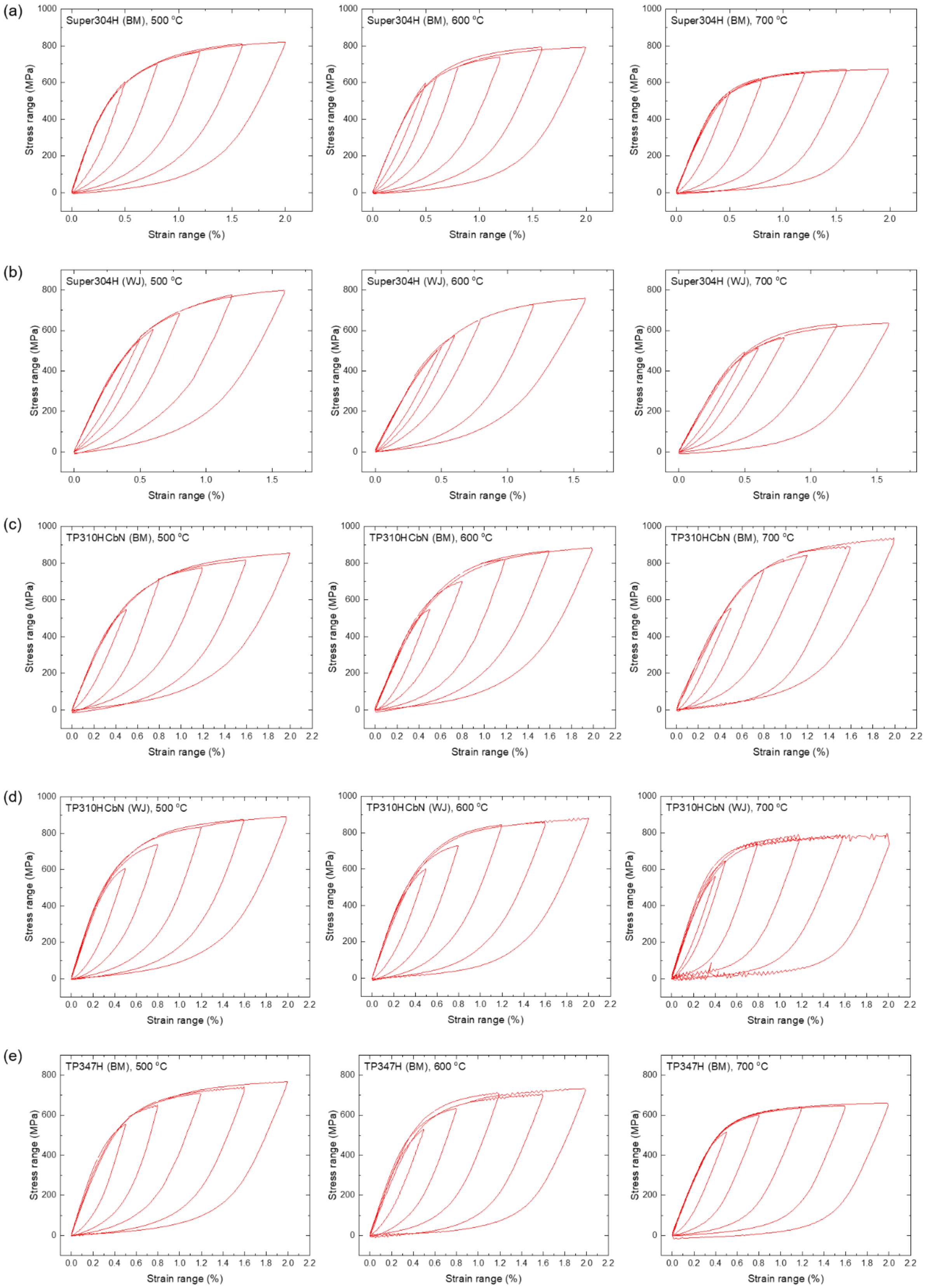
(a)
Super304H (BM), 500 °C
Super304H (BM), 600 °C
Super304H (BM), 700 °C
(b)
Super304H (WJ), 500 °C
Super304H (WJ), 600 °C
Super304H (WJ), 700 °C
(c)
TP310HCbN (BM), 500 °C
TP310HCbN (BM), 600 °C
TP310HCbN (BM), 700 °C
(d)
TP310HCbN (WJ), 500 °C
TP310HCbN (WJ), 600 °C
TP310HCbN (WJ), 700 °C
(e)
TP347H (BM), 500 °C
TP347H (BM), 600 °C
TP347H (BM), 700 °C
Stress range (MPa)
Strain range (%)

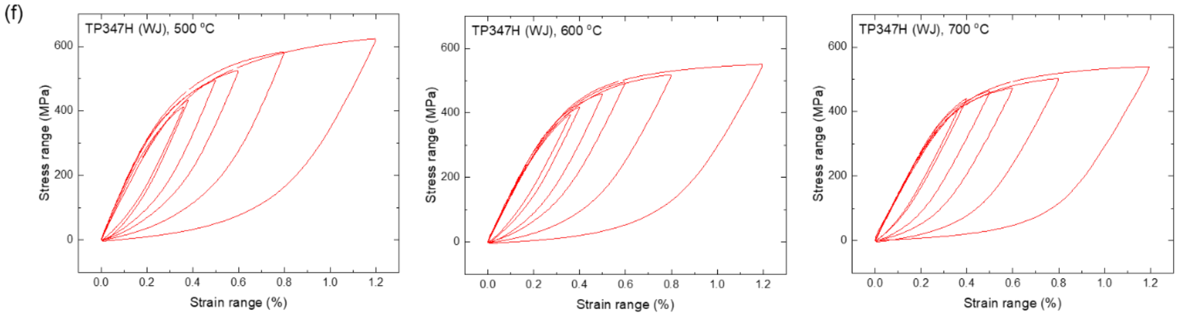

Figure S1. Masing behavior of stabilized stress–strain hysteresis loops at half-life for three base metals and their welded joints at temperatures of 500, 600, and 700 °C. (a) Super304H base metal, (b) Super304H welded joint, (c) TP310HCbN base metal, (d) TP310HCbN welded joint, (e) TP347H base metal, and (f) TP347H welded joint.
